# Evaluation of Nestin and EGFR in Patients with Glioblastoma Multiforme in a Public Hospital in Iran

**DOI:** 10.31557/APJCP.2020.21.10.2889

**Published:** 2020-10

**Authors:** Amir Hassan Matini, Mohadeseh Mofidi Naeini, Hamed Haddad Kashani, Zarichehr Vakili

**Affiliations:** 1 *Department of Pathology, Kashan University of Medical Sciences, Kashan, Iran. *; 2 *Anatomical Sciences Research Center, Institute for Basic Sciences, Kashan University of Medical Sciences, Kashan* ^*, *^ *Ir*an.

**Keywords:** Nestin, angiogenesis, glioblastoma multiforme, microvascular density

## Abstract

**Introduction:**

Glioblastoma multiforme (GBM) is a grade IV glioma and accounts for 15% of all primary brain tumors. This GBM has a median survival range of less than 2 years after diagnosis and it is highly vascularized by neoformed vessels. Neoangiogenesis is a crucial factor in the malignant tumoral behavior and prognosis of patients and Nestin protein belongs to class VI which is expressed in endothelial cells of neoformed vessels in GBM. Our study shows the correlation between EGFR mutation and Nestin expression in endothelial of neoformed vessels in GBM.

**Methods:**

We analyzed 40 GBM samples by immunohistochemistry staining. The immunohistochemical expression of EGFR in tumoral cells and Nestin in endothelial cells in paraffin sections were analyzed. EGFR scoring was the based on staining intensity. Score 0 shows No staining, Score1, mild to moderate staining and score2 sever staining. Microvascular density (MVD) was evaluated with Nestin-immunoreactive.

**Results:**

The mean of MVD was 14.6 ±8.25. Nestin-MVD was significantly higher in GBM with sever vascular prolifration (p-value=0.01). EGFR was expressed in 92.5% of samples. The EGFR scoring for tumoral tissue was 7.5%(score:0), 22.5% (score:1) and 70% (score:2). There was a significant relationship between EGFR expression and MVD (p-value=0.017).

**Conclusion:**

We suggest that some important mutations as like as EGFR in GBM is responsible for inducing angiogenesis and vascular proliferation. Nestin overexpression as a novel marker might reflect the extent of neoangiogenesis, thus target therapy against EGFR pathway and anti angiogenic may be useful for GBM treatment.

## Introduction

According to the American tumor association, brain tumors with prevalence of 12.8 per 100,000 are the most common tumors in the world and Gliomas are the most abundant. Gliomas are the brain parenchymal tumors which are similar in histology to different types of glial cells. The main types of gliomas are astrocytoma and oligodendrogliomas. Astrocytoma has different types that fibrillaryand pilocytic astrocytoma are the most common (Smith et al., 2012). World health organization (WHO) has divided these tumors in to 4 grades based on their cellularity, mitosis, necrosis and vascular proliferation (Pallini, Ricci-Vitiani et al., 2008; McNamara et al., 2013; Moghaddam et al., 2015). The most malignant astrocyte tumors is glioblastoma multiforme (GBM) (Batash et al., 2017). The survival of patients is approximately 12-15 months and less than 3% of patients live more than 5 years(Pallini et al., 2008; McNamara et al., 2013). This tumor is common at age 45-50 years and including 9% of children brain tumors (McNamara et al., 2013). GBM histologically is a heterogeneous tumor consisting of glioma tissue and numerous vessels (Schmidt et al., 2002; Tena-Suck et al., 2015). Although all glioblastoma are in one histological grade, the genetic changes are different (Simmons et al., 2001; Pallini et al., 2008; Liu et al., 2015). One of them is a mutation in Epidermal growth factor receptor (EGFR) gene (Nagpal et al., 2006; Lebelt et al., 2008; Liu et al., 2015). EGFR is a tyrosine kinase receptor which regulates cellular growth and differentiation. Amplifying (40 %<) and over expression (60%>) of EGFR are considerable points in GBMs (Nagpal et al., 2006; Chinnaiyan et al., (2008); Lebelt et al., (2008). GBM has great ability in angiogenesis and proliferation and endothelial hyperplasia among the brain tumors. In fact, angiogenesis is a biological key and an important diagnostic marker for GBM (Ishiwata et al., 2011; Krupkova et al., 2011; Hardee and Zagzag 2012; Tena-Suck et al.. 2015). Angiogenesis is a complicated process in which endogenous markers, chemical signals and changes in the endothelial progenitor cells and surrounding stroma, stimulate and proliferate endothelial cells. In fact, angiogenesis has a key role in cancer growth and development (Kitai et al., 2010; Ishiwata et al., 2011). Angiogenesisin GBM may happen in response to the hypo oxidation in cells of tumor tissue. So, we can usually see proliferation around the necrotic areas(Ishiwata et al., 2011; Sasmita et al., 2018). But there are some evidences indicating that in addition to hypo oxidation, there are other mechanisms, such as mutation in p53, EGFR and growth factors independent to hypo oxidation such as VEGF genes that can lead to angiogenesis(Mokrý et al., 2004; Kitaiet al., 2010; Matsuda et al., 2013). According to the relation between angiogenesis and invasion andprognosis of GBM, study of angiogenesisusing vascular markers in neformed vessels can help with the diagnosis and prognosis of patients. Furthermore inhibition treatments against these markers, can prevent tumor angiogenesis and developments (Shih and Holland 2006; Veselska et al., 2006; Jin et al., 2013). Nestin is a class VI intermediate filaments that expresses in proliferative neuroepithelium during the embryonic development (Shih and Holland 2006; Loja et al., 2009; JR et al. 2010, Dahlrot, Hansen et al., 2014). In adults, Nestin is expressed in subventricular zone where neurogenesis occurs. In addition, Nestin expresses in growing endothelial progenitor cells but not in the matured endothelial cells. So Nestin expression is restricted to neoformed vessels and is more specific than the other markers (Shih and Holland, 2006). In fact, evaluation of the expression of this marker basedon the nuclear staining in immunohistochemistry (IHC) technique can lead to evaluation of micro vascular density (MVD) in tumor surface (Kitai et al., 2010; Ishiwata et al., 2011; Sica et al., 2011; Guadagno et al., 2016). 

## Materials and Methods

40 samples of GBM patients of Shahid Beheshti Hospital, Kashan, Iran were analyzed. The samples belonged to the years 2006-2016. Before selecting, the report on each sample and their H&E slides were reviewed by pathologist. Then, 2 slides from each 5 micron paraffin section were made and stained by immunohistochemistry(IHC) staining using Nestin and EGFR markers. Micro vascular density (MVD) was evaluated by Nestin-immunoreactive. 

Nestin protein expression was detected by Nestin antibody (10c2) (Santa Cruz Biotechnology, Inc.) as a yellow to brown color in the nucleus of vascular endothelial cells. Also the expression of EGFR in nucleus of tumoral cells was evaluated by observing yellow to brown color . In order to determine the Micro Vascular Density (MVD), at first, high density areas (Hot Spot) was determined in low magnification (×40) and then they were counted in high magnification (×400). Each endothelial cell relating to a hot spot was counted as a vessel and the mean of counted vessels in four fields were considered as the Nestin vascular density (Moghaddamet al., 2015). Anti-EGFR specially stains cell membrane and sometimes cytoplasm of cells. Scoring was based on the staining intensity. Score 0 was considered for no staining, score1 for mild to moderate staining and Score 2 for severe staining.

*Statistical analysis*

Data were analyzed by SPSS version 17.0 software. Statistical tests such as chi-square and Fisher’s exact test and odds ratio (OR) were used. P-Value was considered below 5%.

*Ethical considerations*

The case study in this study is tissue sections. Each sample in the pathology laboratory has specific code and its sections has the same code. So at first, the samples were studied without any information. Then further information was gotten from the patients and analyzed. After making required slides, sections were returned to the laboratory.

## Results

Necrosis and Vascular proliferation were observed as an important diagnostic marker in 90% of GBMs. Cellular atypia and pleomorphism were observed in all the tumors in which 17.5% showed mild atypia, 47.5% moderate atypia and 35% severe atypia (Table 1).

**Figure 1:**
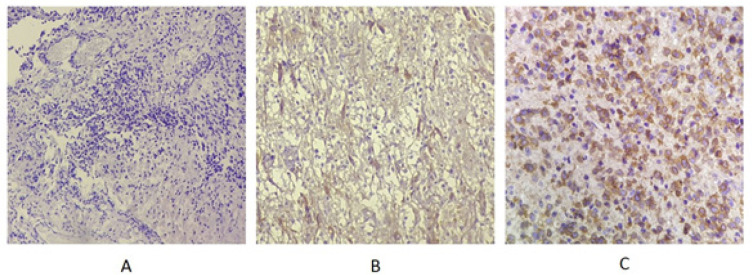
EGFR Marker Staining. A) Score 0, no staining; B) Score1, mild staining; C) Score2, severe staining

**Figure 2:**
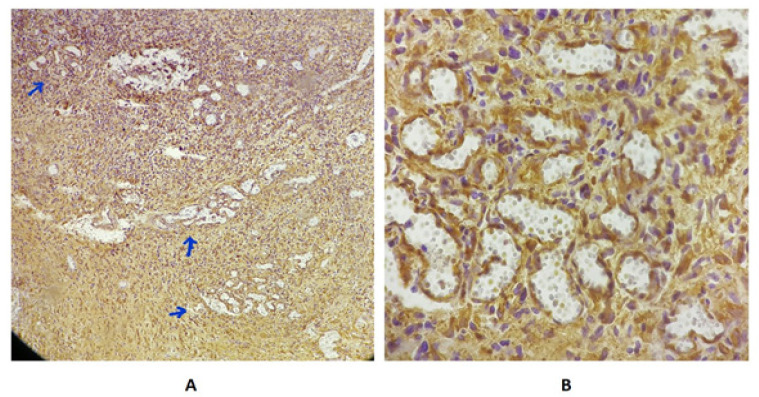
MVD Evaluating Using Nestin Marker. A) High density areas or Hot spots in low magnification (×40). Each Blue arrow indicates a hotspot. B) High density areas in high magnification (×400)

Anti-EGFR staining as the EGFR mutation marker in malignant cells was negative in 3 patients and was positive in 37 patients. 8 patients showed score 1 and 29 patients showed score 2. The frequency for score 0 was 3 (7.5%), for score 1 was 9 (22.5%) and for score 2 was 28 (70%) (Figure 1). The mean of Micro Vascular Density (MVD) using Nestin marker in all samples was 14.6± 8.25 (Figure 2).

**Figure 3:**
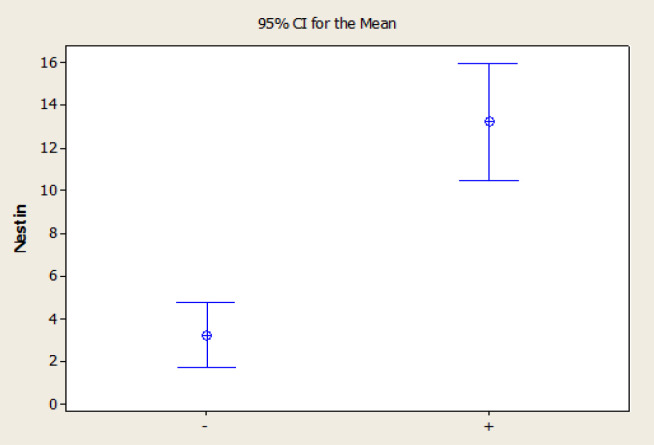
The Mean of Micro Vascular Density in Presence or Absence of Vascular Proliferation

The mean of MVD in the group with vascular proliferation was 13.22 ±8.09 and in the group without proliferation was 3.25 ±0.96. The difference between two groups was significant (P-value=0.01) (Figure3).

**Figure 4:**
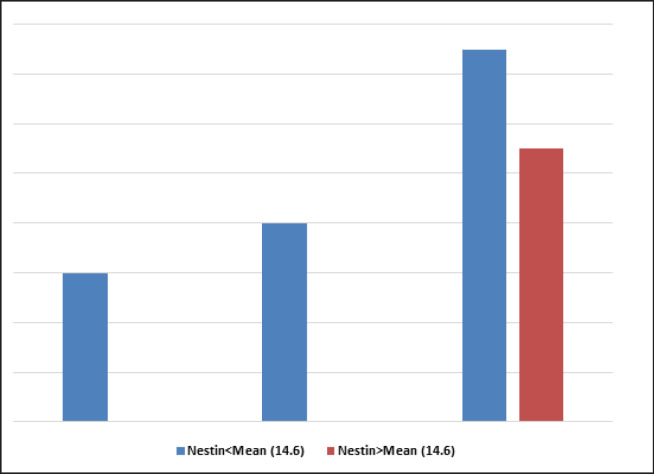
MVD Frequency and EGFR Expression

**Figure 5:**
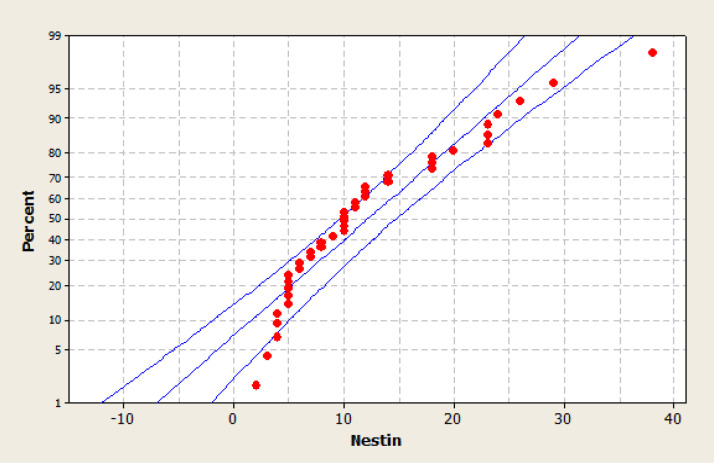
MVD and Nestin Expression

Frequency in the group with no EGFR expression (Figure 4) in which MVD was below themean, was 6, and with mild to moderate expression was 8 and with severe expression was 15. Frequency in the group in which MVD was above the mean, just observing in severe EGFR expression, was 11. There was significant relation between MVD and EGFR expression and P_value was 0.017 (Table 2). However, the MVD and Nestin showed an increased expression (Figure 5).

**Table 1:** Pathological Features of Tumors

Pathologic features	Frequency
Tumors with Necrosis	36 (90%)
Tumors without Necrosis	4 (10%)
Tumors with vascular proliferation	36 (90%)
Tumors without vascular proliferation	4 (10%)
Tumors with mild atypia and poleomorphism	7 (17.5%)
Tumors with moderate atypia and pleomorphism	19 (47.5%)
Tumors with severe atypia and pleomorphism	14 (35%)

**Table 2:** The Relation between EGFR Expression and MVD

		EGFR Expression		
		Score 0	Score 1	Score 2
MVD	Below the mean (<14.6)	6 (15%)	8 (20%)	15 (38%)
	Above the mean (>14.6)	0	0	11 (27%)

## Discussion

Available treatments for GBM such as surgery, radiation and chemotherapy cannot survive the patients more than 14 months. So development of treatment approaches is an important issue (Cho et al., 2011). Although GBM is prevalent in patients of all ages, it is more observed in the elderly and in white men. 

Jazayeri et al., (2013) reported that the mean age of GBM patients is 50.6±16.9 in Iran and the incidents has been reported 0.79 case per 100,000 in years 2000-2010 (Jazayeri et al., 2013). 

In our study the mean age of patients was 53.32±16.04 at the range of 18-88 years and it was more common in males. Necrosis, cellular atypia and vascular proliferation are the most important diagnostic factors in GBM. 

Tena-Suck et al., (2015) have reported that necrosis was founded in 66% of primary tumors and 82% of secondary tumors. There were more vascular proliferation in more inflamed and necrotic tumors but there was no significant correlation (Tena-Suck et al., 2015).

In this study, necrosis was observed in 90% of samples but there was not any correlation between necrosis and age and vascular proliferation. However Cellular atypia and pleomorphism were found in all samples and were significantly associated with necrosis.

One of the important diagnostic factor in GBM is angiogenesis. So evaluation of tumor vascular density and anti-angiogenic therapies can play an important role in the patient’s prognosis and recovery. Several studies have been conducted on micro vascular density (MVD) of tumorvessels using various vascular markers such as CD31 and CD34. These markers can stain both small and large vessels with the same intensity. In addition they are in both tumor and normal tissue vessels (Guadagno et al. 2016). Nestin is a class VI intermediate protein that expresses in malignant cells of GBM and can stain neoformed vessels. So it is a good marker for MVD evaluating (D’Alessio et al. 2016). A few studies have been performed on the MVD using Nestin and the angiogenesis process in GBM. 

Chinnaiyan et al., (2008) and D’Alessio et al., (2016) have separately reported that Nestin expresses in the GBM stem cells and neoformed vessels endothelial. So in addition to the proliferation of tumor cells, it also stimulates the angiogenesis process. 

In our research, the expression of Nestin in the vessels of malignant cells is related to the vascular proliferation in GBM. MVD in the areas with vascular proliferation is about 13.2. So Nestin is a sensitive marker for evaluating MVD. 

Krupkova et al., (2006) have found that Nestin expression is related to the tumor grade and it is over expressed in high grade tumors. So it is a good candidate for GBM diagnosis (Veselska et al., 2006). In this study, there was no correlation between Nestin expression and necrosis or cellular atypia. Genetic mutations have a key role in forming GBMs. EGFR is an essential growth factor in epithelial tissues and abnormal signaling lead to the formation of epithelial malignity. It has been proposed that genetic mutation of EGFR gene is responsible for the pathogenesis of gliomas. Mechanisms for mutation of EGFR in malignity include structural rearrangements of the receptor, EGFR gene amplification, activating mutations in the EGFR kinase domain and overexpression of (EGF)–family ligands by tumor cells and/or surrounding stroma (Arif et al., 2015, Alamdari-Palangi et al., 2020).

Schmidt et al., (2002) had a study on the glioblastoma samples in 2002 and indicated that EGFR gene amplification in less than 40% cases and overexpression in more than 60% cases are a remarkable point in GBM. These events are associated with increased invasion, adhesion, malignant cells proliferation and inducing of vascular proliferation (Simmons et al., 2001; Talasila et al., 2013). 

In new GBM treatments approaches, it has been focused on the tyrosine kinase inhibition and growth factors-related pathways. Over activity of EGFR pathway is associated with resistance to the chemotherapy and radiotherapy. Therefore target therapy and targeted chemotherapy increase treatment effects (Arif et al., 2015). 

In this study, expression of EGFR marker observed in 92.5% of cases in which 22.5% of cases have score1 and 70% of cases have score2. But there isn’t any correlation between EGFR expression and age, necrosis, vascular proliferation and cellular atypia. 

Talasila et al., (2013) indicated that although severe mutation of EGFR in GBM is associated with non-vascular invasion, it can provide vascular proliferation on long term. In this study, we evaluated the rate of EGFR mutation in GBM and MVD, especially in neoformed vessels, using Nestin marker and the relation between angiogenesis and EGFR mutation. There is a positive correlation between MVD and EGFR mutation. It shows that mutations such as EGFR mutation, in addition to the tumor cells’ proliferation can affect angiogenesis induction (Eskilsson et al., 2018). 

In conclusion, we conclude that both EGFR mutation and inhibition of vascular proliferation are the remarkable therapeutic targets in GBM. So combination therapy against them can be effective in patients’ prognosis. Further studies about the association between these targets are needed to find new diagnostic and therapeutic approaches for GBM. 

## Data Availability

The dataset used in this study is available with the authors and can be made available upon request.

## Abbreviations

WHO: world health organization

MVD: MicroVascular Density

EGFR: Epidermal Growth Factor Receptor

H&E: Hematoxylinand Eosin

GBM: Glioblastoma Multiforme

VEGF: vascular endothelia growth factor

IHC: immunohistochemistry
